# Choosing wisely? Quantifying the extent of three low value psychotropic prescribing practices in Australia

**DOI:** 10.1186/s12913-018-3811-5

**Published:** 2018-12-29

**Authors:** Jonathan Brett, Helga Zoega, Nicholas A. Buckley, Benjamin J. Daniels, Adam G. Elshaug, Sallie-Anne Pearson

**Affiliations:** 1 Medicines Policy Research Unit, Centre for Big Data Research in Health, University of New South Wales, Sydney, NSW 2052 Australia; 2 Centre of Public Health Sciences, Faculty of Medicine, University of Iceland, Reykjavik, Iceland; 3 School of Medicine, The University of Sydney, Sydney, NSW Australia; 4 Menzies Centre for Health Policy, The University of Sydney, Sydney, Australia

**Keywords:** Elderly, Benzodiazepines, Antipsychotics, Dementia, Polypharmacy, Choosing wisely

## Abstract

**Background:**

The global Choosing Wisely campaign has identified the following psychotropic prescribing as low-value (harmful or wasteful): (1) *benzodiazepine use in the elderly*, (2) *antipsychotic use in dementia* and (3) *prescribing two or more antipsychotics concurrently*. We aimed to quantify the extent of these prescribing practices in the Australian population.

**Methods:**

We applied indicators to dispensing claims of a 10% random sample of Australian Pharmaceutical Benefits Scheme beneficiaries to quantify annual rates of each low-value practice from 2013 to 2016. We also assessed patient factors and direct medicine costs (extrapolated to the entire Australian population) associated with each practice in 2016.

**Results:**

We observed little change in the rates of the three practices between 2013 and 2016. In 2016, 15.3% of people aged ≥65 years were prescribed a benzodiazepine, 0.5% were prescribed antipsychotics in the context of dementia and 0.2% of people aged ≥18 years received two or more antipsychotics concurrently. The likelihood of elderly people receiving benzodiazepines or antipsychotics in the context of dementia increased with age and the likelihood of receiving all three practices increased with comorbidity burden. In 2016, direct medicine costs to the government of all three practices combined, extrapolated to national figures, were > $21 million AUD.

**Conclusions:**

Our indicators suggest that the frequency of these three practices has not changed appreciably in recent years and that they incur significant costs. Worryingly, people with the greatest risk of harm from these prescribing practices are often the most likely to receive them.

**Electronic supplementary material:**

The online version of this article (10.1186/s12913-018-3811-5) contains supplementary material, which is available to authorized users.

## Background

The conception of the global Choosing Wisely campaign has heralded a renewed interest in addressing low-value care; medical practices such as test ordering, procedures or prescribing that are either harmful or wasteful [1]. The Choosing Wisely campaign is a physician-led, grass-root program which generates evidence-based ‘top 5 lists’ of practices considered low-value across a breadth of medical specialties [2, 3]. Choosing Wisely was conceived in the U.S. in 2012 and has since spread globally across Europe and Asia to over 20 countries around the world, launching in Australia in April 2015 [4]. The campaign nominates low-value practices to be focal points of conversations between patients and physicians with the ultimate aim of promoting alternative, more appropriate care. Developing indicators to quantify the extent of low-value medical practices allows policy priorities to be set and any changes following quality improvement strategies to be measured.

The majority of previous population-based research on low-value care has focused on medical tests and procedures [5], with limited attention to low-value prescribing practices [6]. This is despite the fact that when the nominated low-value practices in each country participating in the Choosing Wisely campaign are pooled, prescribing practices constitute nearly one quarter of all practices [6]. Of these, psychotropic prescribing practices feature heavily, and while measuring inappropriate psychotropic use in routinely collected data is not novel, examining them through the lens of low-value care is a relatively recent endeavor.

Most efforts to quantify low-value prescribing to date, such as the Australian Atlas of Variation in Health Care [7], have been based on aggregated dispensing claims data. Within the constraints of data content, individual-level analyses enhance measurement approaches by allowing individuals receiving low-value prescribing practices to be more accurately identified.

Our overall objective was to develop indicators that can be applied to Pharmaceutical Benefits Scheme (PBS) data to quantify three low-value psychotropic prescribing practices in Australia. These practices were chosen as they were mentioned repeatedly within and between pooled national Choosing Wisely lists [6] and were conducive to measurement approaches using dispensing claims data. These practices are: (1) *don’t use benzodiazepines in the elderly*, (2) *avoid antipsychotics for dementia* and (3) *don’t routinely prescribe two or more antipsychotics concurrently*. Specifically, we applied indicators to estimate the annual rates of these practices between 2013 and 2016 and their association with patient age, sex and co-morbidity. While this may not capture changes resulting from the launch of Choosing Wisely in Australia, it provides an understanding of any underlying trends. We also estimated the direct costs of the medicines involved in each low-value psychotropic prescribing practice to the Australian government and patients.

## Methods

### Study setting, data and population

Australia has a publicly funded universal healthcare system entitling all citizens and permanent residents to subsidised access to prescription medicines via the Pharmaceutical Benefits Scheme (PBS). In this study, we used PBS dispensing records from 2010 to 2016 for a 10% random sample of all persons eligible to receive PBS-listed medicines. This is a standard dataset provided by the Australian Government Department of Human Services for analytical use and captures the majority of community based medicine dispensing [8]. Our study population consisted of all people 18 years and older within the PBS 10% sample with at least one dispensing record during the study period, 1 January 2013 through 31 December 2016.

### Medicines of interest

We included medicines belonging to Anatomical Therapeutic Chemical Classification (ATC) classes antipsychotics (NO5A), benzodiazepines (NO5BA, NO5CD and NO3AE) and anti-dementia medicines (NO6D) [9] that were PBS-subsidised in Australia during the study period (see Additional file 1: Table S1 for full details of medicines and ATC codes).

### Indicators of low-value prescribing practices

Since some measures require specific clinical information that is not contained within available dispensing claims data, we developed primary and secondary indicators for each low-value practice to illustrate the sensitivity of measurement to indicator definition (Table 1).

**Table 1:** Primary and secondary indicators applied to Pharmaceutical Benefits Scheme (PBS) dispensing claims data to measure each low-value prescribing practice

Low-value practice	Indicator		
	Primary	Secondary	Secondary indicator justification
*Don’t use benzodiazepines in the elderly*	People ≥65 years with prevalent benzodiazepine use	People ≥65 years with incident (new) benzodiazepine use	New benzodiazepine use defines a narrower population of recipients of this practice than prevalent use
*Avoid antipsychotics for dementia*	People ≥65 years dispensed an anti-dementia medicine in the same year or the three calendar years prior to being dispensed an antipsychotic^a^	People ≥65 years with incident (new) antipsychotic use	Incident antipsychotic use is unlikely to be for an indication where the evidence of risk and benefit is clear (schizophrenia or bipolar disorder)
*Don’t routinely prescribe two or more antipsychotics concurrently*	People ≥18 years dispensed two or more antipsychotics with overlapping treatment exposures of 60 days	As for primary but excluding long acting depot preparations^b^	Concomitant use of a long acting depot with an oral antipsychotic may be necessary during initial stages of therapy while dosing is being optimized

^a^Anti-dementia medicines are PBS subsidised for Alzheimer’s dementia but may be used in other forms of dementia in practice [34]

^b^Long acting depot preparations are identified in Additional file 1: Table S1

In this study, we defined annual incident (new) use as a dispensing of a medicine in a given year with no dispensings of that medicine in the 12 months prior and annual prevalent use as any dispensings of that medicine in a given year. We defined concurrent use of two or more antipsychotics (hereafter referred to as antipsychotic polypharmacy) as an overlap in antipsychotic exposure of > 60 days; an intentionally conservative measure that has been validated in dispensing claims data [10, 11]. The duration of exposure following a single antipsychotic dispensing was estimated as the time by which 75% of people had a repeat dispensing the same antipsychotic as previously described [10].

### Data analysis

#### Annual rates of low-value psychotropic prescribing (2013–2016)

We applied each indicator to estimate the number of persons experiencing the corresponding low-value practice in each calendar year. We divided this by the corresponding mid-calendar year population estimates from the Australian Bureau of Statistics (ABS) [12] for people in the relevant age group to calculate the annual rates of each prescribing practice.

We used different denominators to illustrate changes from an indication perspective [13] and for our primary indicators expressed antipsychotic use in the context of dementia as a percentage of people with dementia (defined as people with a prior anti-dementia medicine dispensing) and antipsychotic polypharmacy as a percentage of people receiving any antipsychotic.

#### Patient factors associated with low-value prescribing practices

For our primary indicators only, we stratified the rates of each low-value practice in 2016 by patient age, sex and comorbidity score. We categorised age at the time of dispensing as 65–74, 75–84 and ≥ 85 years for benzodiazepines in the elderly and antipsychotics in the context of dementia and as 18–49, 50–64 and ≥ 65 years for antipsychotic polypharmacy. We estimated the number of comorbidities for each individual in the population dispensed any medicine with the RxRisk score [14] using the 12 months of dispensings prior to the first dispensing of any medicine in 2016. For each practice, we excluded comorbidities from the RxRisk score that were part of the practice to avoid inflating associations and categorised the number of comorbidities as; 0–2, 3–5, 6–8, and ≥ 9.

To estimate the association of patient age, sex and comorbidity score with each low-value practice indicator in 2016, we ran crude and adjusted logistic regression analyses. We expressed associations as crude and adjusted odds ratios with 95% confidence intervals, using males, the youngest age group and 0–2 comorbidities as reference categories.

#### Direct medicine costs

For our primary indicators only, we calculated direct medicine costs by identifying all dispensings associated with each low-value practice in 2016. For antipsychotic polypharmacy we summed the costs of all antipsychotics involved in a polypharmacy episode, as we were not able to determine appropriate monotherapy. We calculated medicine costs to the Australian government and patients based on co-payment thresholds and medicine cost at the date of dispensing using historical data supplied by the PBS Advisory Board. To obtain national cost estimates we multiplied these estimates from the PBS 10% sample by ten.

All analyses were performed with SAS, version 9.4 (SAS Institute Inc) and Stata version 12 (Statacorp).

## Results

**Fig. 1:**
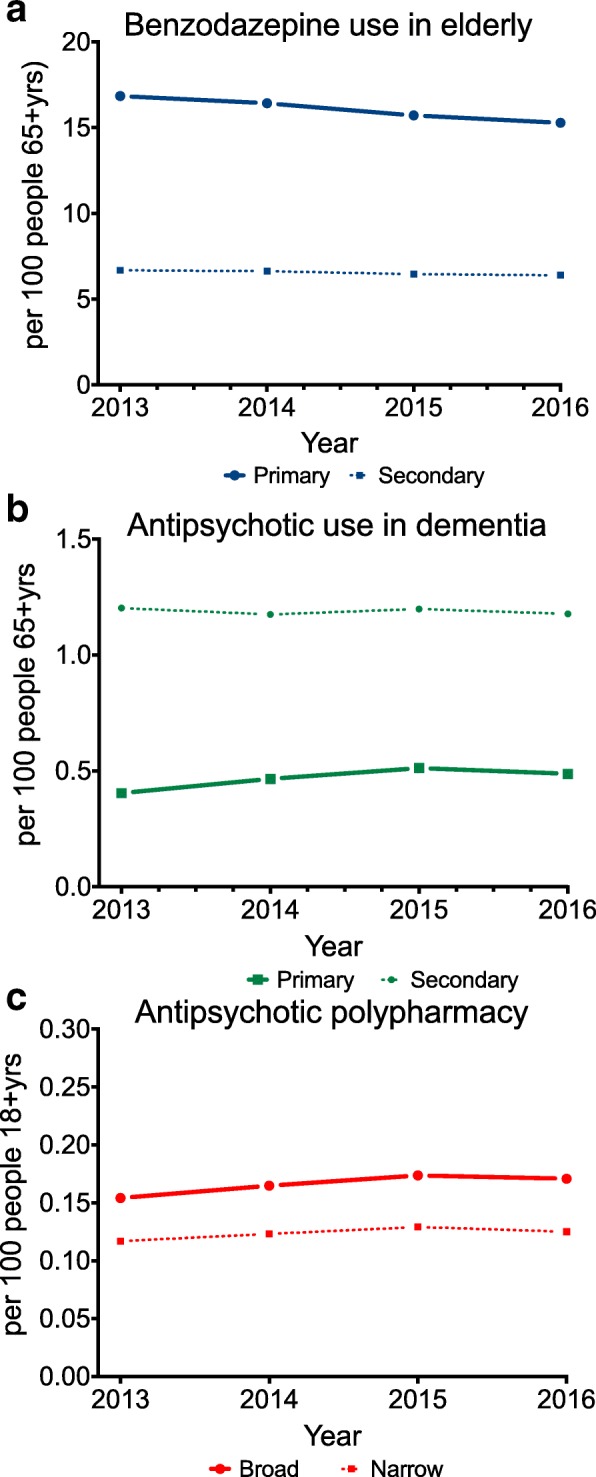
Annual rate of low-value prescribing practice indicators: primary (solid) and secondary (dash); **a** benzodiazepine use in the elderly (primary = prevalent use, secondary = incident use) **b** antipsychotic use in dementia (primary = past anti-dementia medicine, secondary = incident antipsychotic use), **c** antipsychotic polypharmacy (primary = all antipsychotic polypharmacy, secondary = antipsychotic polypharmacy minus long acting depots)

According to our indicators, we found little to no change in the annual rates of each low-value prescribing practice across the study years. The rates of both primary and secondary indicators are shown in Fig. 1 but henceforth we refer only to primary indicators. The rate of prevalent use of benzodiazepines in the elderly (≥65 year olds) was 16.5% in 2013 and 15.3% in 2016. Antipsychotic use in the context of dementia in the elderly was 0.4% in 2013 and 0.5% in 2016 and the rate of antipsychotic polypharmacy remained 0.2% throughout the study period.

Among people with dementia, 24.9% received an antipsychotic in 2013 and 21.3% in 2016. Among people who were dispensed an antipsychotic, 6.7% received antipsychotic polypharmacy in 2013 and 7.1% in 2016 (Additional file 1: Figure S1a and b).

### Patient factors associated with low-value psychotropic prescribing

**Fig. 2:**
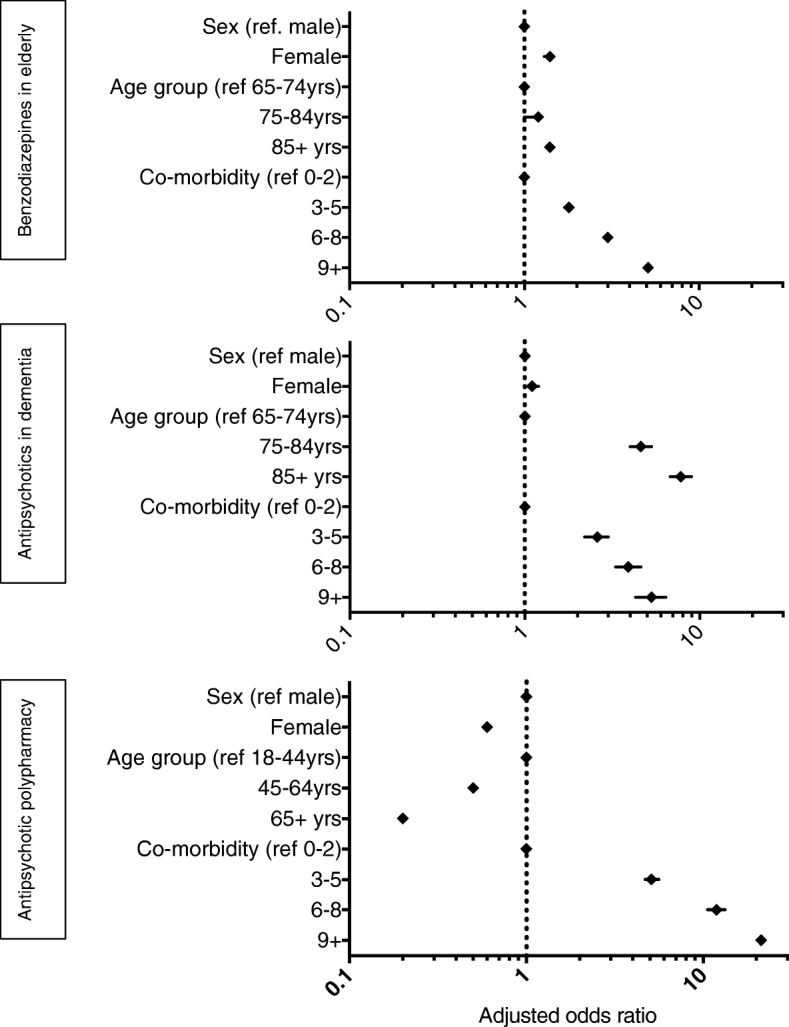
Adjusted odds ratios from multiple logistic regression analyses for patient factors associated with each low-value prescribing practice in 2016

In 2016, based on primary indicators; elderly women had greater adjusted odds than men of receiving benzodiazepines and the adjusted odds of receiving a benzodiazepine increased with patients’ age and number of comorbidities (Fig. 2, Additional file 1: Table S2). The adjusted odds of receiving an antipsychotic in the context of dementia were independent of sex but increased with age and comorbidity. The adjusted odds of receiving antipsychotic polypharmacy decreased with age and increased with comorbidity (Fig. 2, Additional file 1: Tables S2, S3 and S4 for adjusted and crude odds ratios as well as stratified prevalence of each practice).

Based on the extrapolated costs of our primary indicators, the direct medicine cost of benzodiazepine use in the elderly in 2016 was $13.8 million to patients and $12.2 million to the government; for antipsychotic use in the context of dementia extrapolated costs were $0.7 million to patients and $2.1 million to the government; and for antipsychotic polypharmacy costs were $0.5 million to patients $5.3 million to the government (Table 2).

**Table 2:** Number of dispensings and extrapolated direct medicine costs of three low-value prescribing practices in 2016

		Low-value prescribing practice		
		Benzodiazepines in elderly	Antipsychotics in dementia	Antipsychotic polypharmacy
Number of dispensings (in PBS 10% sample)		240,460	11,777	51,310
Extrapolated direct medicine costs (/$ million)	Patients	13.8	0.7	0.5
	Government	12.2	2.1	5.3
	Total	26	2.8	5.8

## Discussion

Here we developed indicators within PBS dispensing claims to describe annual trends, associated patient factors and direct medicine costs of three low-value prescribing practices as nominated by the Choosing Wisely campaign. According to these indicators, there were no major changes in the rate of any of these practices on a national level over the study period. This is despite long-standing evidence of the poor risk-benefit profiles of the practices described [15–17] and multiple national and local clinical interventions targeting these practices during this time [18]. There may however have been changes in the rates of these practices within smaller geographical areas that we did not examine.

While it may be too early to detect a measurable impact, according to our indicators, there has been no discernable change in the rates of these practices in relation to the launch of the Choosing Wisely Australia campaign in April 2015. However, only benzodiazepine use in the elderly and antipsychotic use in dementia were mentioned in the Choosing Wisely Australia campaign [19] and the wording of these practices specified indications and/or caveats. For instance the Australian and New Zealand Society of Geriatric Medicine states “do not prescribe benzodiazepines or other sedative-hypnotics to older adults as first choice for insomnia, agitation or delirium” and “do not use antipsychotics as the first choice to treat behavioral and psychological symptoms of dementia (BPSD)”. Despite this it is likely that our measurement of benzodiazepine use in the elderly closely approximates the Australian variant of this practice, as benzodiazepines make up almost all sedative-hypnotic use in the elderly population. Furthermore, with the exceptions of epilepsy (clonazepam only) and anxiety, there would be few other indications that they would be used for in the elderly other than insomnia, agitation or delirium [20].

Caveats within these practices exemplify challenges inherent to measuring many Choosing Wisely recommendations in routinely collected data and suggest that quantification of these practices might not have been considered when lists of low-value practices were being constructed. For instance, it is difficult to determine whether antipsychotics are being used as ‘first choice’ in dementia when the preferred first choice is a non-pharmaceutical intervention that is not recorded in dispensing claims data. Similarly, don’t routinely prescribe two or more antipsychotics concurrently also begs the question of how frequent ‘routinely’ actually is and on a clinical level this is open to inconsistent interpretation. Here we quantify prescribing practices defined as low-value a-priori by the Choosing Wisely campaign, and while there may be clinical circumstances in which such prescribing is appropriate we have taken these practices at face value. Historically, a number of other physician-led, consensus-based guidelines such as the Beers and STOPP criteria [21, 22] have been developed to identify potentially inappropriate prescribing practices and assist with de-prescribing, but these tend to focus on specific populations (such as the elderly).

Other measurement issues related to ascertainment become apparent when comparing this study to U.S. studies of antipsychotic use in dementia using Medicare data where dementia diagnoses from clinical encounter information are available. One such study showed mean annual rates of antipsychotic use in dementia to be higher (31%) than our study (21% in 2016), most likely because of more accurate dementia ascertainment [23]. Another UK study found that 15.2% of elderly patients with dementia had received an antipsychotic, but this study only included people prescribed ‘low doses’ within one primary care trust [24].

It is concerning, but not surprising, that the likelihood of receiving the first two practices increases with age, and the likelihood of receiving all three practices increases with comorbidity burden, as this is accompanied by a commensurate increase in the risk of adverse events associated with frailty, polypharmacy and drug-disease interactions [25]. Hence, caution is required when prescribing antipsychotics in this clinical context.

While the main focus of the Choosing Wisely campaign is to promote safe and effective care it is clear that low-value prescribing also incurs significant direct medication costs, totaling $21 million AUD to the government in 2016. However, the majority of the cost of these practices is likely to be related to their clinical adverse effects [6]. For example, Table 3 illustrates how the cost to the government of excess hip fractures related to benzodiazepine use in the elderly may be in the region of $29 million AUD annually. However, these potential cost savings do not account for the additional costs of alternative non-pharmacological treatments. This one adverse drug event, when added to direct medicine costs brings the total cost of benzodiazepine use in the elderly to $41 million.

**Table 3:** Calculation of downstream costs of benzodiazepine use in the elderly related to hip fractures

Estimated adjusted rate ratio of hip fractures in elderly people (≥50 years) taking benzodiazepines compared to those who were not = 1.52 (95% CI: 1.37-1.68) [23] Proportion of elderly people (≥65 years) dispensed benzodiazepines (current study) = 0.067 Estimated number of osteoporotic & osteopenic hip fractures in 2016 = 26,232 [34] Estimated average direct cost of a hip fracture in 2012 = $32,569 AUD [34] Proportion of hip fractures attributable to benzodiazepines $$ =\frac{\left(1.52-1\right)\ast 0.067}{\left[\left(1.52-1\right)\ast 0.067\right)\Big]+1}\kern0.5em =0.033 $$ Number of hip fractures attributable to benzodiazepines = 0.033*26,232 = 883 Excess costs of hip fractures related to benzodiazepine use in the elderly = 883*$32,569 = **$29 million (95% CI: $20-$38 million) AUD**	

## Implications for policy and future research

Our indicators of three low-value psychotropic prescribing practices have demonstrated a lack of any major changes in their frequency, suggesting a more concerted national effort is required to realize decreases in these practices and their related harms. Part of this process must be a deep understanding of the views of prescribers and the pressures they experience as well as synthesizing existing prescribing practice-specific intervention related literature [26]. The indicators developed here could be used to measure changes resulting from any ensuing interventions. Knowledge of patient characteristics associated with these practices also provides some focus for policy interventions. For instance, renewed efforts to target benzodiazepine and antipsychotic use in nursing homes is likely to have the highest yield as an initial approach as this population tends to be older and have more co-morbidity [27–29].

## Limitations

Private prescribing is not captured within PBS dispensing claims and this is estimated to account for up to 10 and 6% of all benzodiazepine and antipsychotic prescribing respectively [30]. We did not include z-drugs in this study as they are not routinely captured in PBS data and they only account for a small proportion of sedative dispensings in Australia [30]. In this study we were unable to capture dose reductions amongst individuals taking these medicines in attempts by prescribers to minimise harm.

Medicines priced beneath the co-payment threshold were not captured within PBS data prior to July 2012 but this unlikely to have affected our results [8]. We made assumptions about the duration of exposure following one dispensing of a medicine in order to determine polypharmacy. Sensitivity analyses around these assumptions, published elsewhere [10] demonstrate that this is unlikely to affect trends but may underestimate the true prevalence of antipsychotic polypharmacy. The direct medicine costs of antipsychotic polypharmacy includes all antipsychotics involved in the polypharmacy episode as without clinical details we were unable to identify appropriate monotherapy. Therefore the cost of antipsychotic polypharmacy will be an overestimate. While the RxRisk score is predictive of mortality, it may not capture all co-morbidities [31]. Past dispensings claims for anti-dementia medicines only capture between 40 and 60% of people with dementia [32, 33], therefore our primary indicator of this practice will most likely under-estimate antipsychotic use in dementia. Applying the broader secondary indicator of people receiving new antipsychotic dispensings is likely to capture all people using antipsychotics in the context of dementia but may also capture antipsychotic use for other conditions such as delirium. We used a three-year look-back to identify past anti-dementia medicine dispensings, as we believe that this will capture most people with a prior dispensing of these medicines.

Finally, clinically contextual information such as the indication for which the medicine was prescribed and comorbidities was not available and hence we cannot be certain that prescribing was inappropriate. However, we have endeavored to quantify these three low-value practices as nominated by the Choosing Wisely campaign rather than to make judgments about the clinical appropriateness of prescribing in each instance.

## Conclusions

The ultimate aim of campaigns such as Choosing Wisely is to optimize patient safety and quality of care. The lack of any substantial change in indicators of three low-value psychotropic prescribing practices described here, along with their relatively high rates and potential cost implications, indicates that renewed efforts are required to achieve further decreases in the rates of these practices.

## Additional file

Additional file 1: **Table S1.** List of included medicines and ATC codes as well as list of item codes excluded to define secondary indicator for antipsychotic polypharmacy. **Table S2.** Low-value psychotropic prescribing practices, adjusted odds ratios of associated patient characteristics and direct medicine costs in 2016. **Table S3.** Crude odds ratios of associated patient characteristics in 2016 for each low-value psychotropic prescribing practice. **Table S4.** Prevalence of each low-value practice in 2016 based on primary indicators by sex and age group. **Figure S1.** Annual rate of low-value prescribing practice indicators; a) Antipsychotic use in dementia (defined by past anti-dementia medicine) b) Antipsychotic polypharmacy (all antipsychotics included). (DOCX 315 kb)

## Abbreviations

ABS: Australian Bureau of Statistics
ATC: Anatomical Therapeutic Chemical Classification
AUD: Australian Dollars
BPSD: Behavioral and psychological symptoms of dementia
PBS: Pharmaceutical Benefits Scheme
