# Supplementary material for: Choosing wisely? Quantifying the extent of three low value psychotropic prescribing practices in Australia
Source: BMC Health Serv Res. 2018 Dec 29;18:1009. doi: 10.1186/s12913-018-3811-5 (PMC6310957; doi:10.1186/s12913-018-3811-5)
Supplement: Supplementary file 1 — Table S1. List of included medicines and ATC codes as well as list of item codes excluded to define secondary indicator for antipsychotic polypharmacy. Table S2. Low-value psychotropic prescribing practices, adjusted odds ratios of associated patient characteristics and direct medicine costs in 2016. Table S3. Crude odds ratios of associated patient characteristics in 2016 for each low-value psychotropic prescribing practice. Table S4. Prevalence of each low-value practice in 2016 based on primary indicators by sex and age group. Figure S1. Annual rate of low-value prescribing practice indicators; a) Antipsychotic use in dementia (defined by past anti-dementia medicine) b) Antipsychotic polypharmacy (all antipsychotics included). (DOCX 315 kb) [file 12913_2018_3811_MOESM1_ESM.docx]

**Additional file 1**

**Table S1:** List of included medicines and ATC codes as well as list of item codes excluded to define secondary indicator for antipsychotic polypharmacy

| **Medicine** | **Class** | **ATC code** | **Item codes excluded depot antipsychotics** |
| --- | --- | --- | --- |
| Alprazolam | Benzodiazepine | N05BA12 | - |
| Diazepam | Benzodiazepine | N05BA01 | - |
| Oxazepam | Benzodiazepine | N05BA04 | - |
| Nitrazepam | Benzodiazepine | N05CD02 | - |
| Temazepam | Benzodiazepine | N05CD07 | - |
| Amisulpride | Antipsychotic | N05AL05 | - |
| Aripiprazole | Antipsychotic | N05AX12 | 10219W, 10224D |
| Asenapine | Antipsychotic | N05AH05 | - |
| Clozapine | Antipsychotic | N05AH02 | - |
| Olanzapine | Antipsychotic | N05AH03 | 9294E, 9295F, 9303P |
| Paliperidone | Antipsychotic | N05AX13 | 11072R, 11094X, 5100K, 5103N, 5107T, 5102M, 11085K, 5109X, 11066K |
| Quetiapine | Antipsychotic | N05AH04 | - |
| Risperidone | Antipsychotic | N05AX08 | 8781E, 8782F, 8780D |
| Ziprasidone | Antipsychotic | N05AE04 | - |
| Chlorpromazine | Antipsychotic | N05AA01 | - |
| Flupenthixol Decanoate | Antipsychotic | N05AF01 | 2257T, 2255Q |
| Haloperidol | Antipsychotic | N05AD01 | - |
| Haloperidol Decanoate | Antipsychotic | N05AD01 | - |
| Pericyazine | Antipsychotic | N05AC01 | - |
| Thioridazine | Antipsychotic | N05AC02 | - |
| Trifluoperazine | Antipsychotic | N05AB06 | - |
| Zuclopenthixol Decanoate | Antipsychotic | N05AF05 | 8097E |
| Donepezil | Anti-dementia medicine | N06DA02 | - |
| Rivastigmine | Anti-dementia medicine | N06DA03 | - |
| Galantamine | Anti-dementia medicine | N06DA04 | - |
| Memantine | Anti-dementia medicine | N06DX01 | - |

**Table S2:** Low-value psychotropic prescribing practices, adjusted odds ratios of associated patient characteristics and direct medicine costs in 2016

| **2016** | **Low-value prescribing practice**^†^ | | |
| --- | --- | --- | --- |
|  | Benzodiazepines in elderly | Antipsychotics in dementia | Antipsychotic polypharmacy |
| **Rate (% people)** | 15.3 | 0.5 | 0.2 |
| **Number of dispensings**  (in PBS 10% sample) | 240,460 | 11,777 | 51,310 |
| **Patient characteristics** |  |  |  |
| **Adjusted**^‡^ odds ratios  (95% CIs) |  |  |  |
| Sex |  |  |  |
| Male | ref | ref | ref |
| Female | 1.4 (1.3-1.4) | 1.1 (1.0-1.2) | 0.6 (0.5-0.6) |
| Age group^\|\|^ |  |  |  |
| 1 | ref | ref | ref |
| 2 | 1.2 (1.1-1.2) | 4.6 (4.0-5.3) | 0.5 (0.5-0.6) |
| 3 | 1.4 (1.4-1.4) | 7.8 (6.8-9.0) | 0.2 (0.1-0.2) |
| Comorbidity (RxRisk) Score |  |  |  |
| 0-2 | ref | ref | ref |
| 3-5 | 1.8 (1.7-1.8) | 2.6 (2.2-3.0) | 5.1 (4.7-5.6) |
| 6-8 | 3.0 (2.9-3.1) | 3.9 (3.3-4.6) | 11.9 (10.6-13.3) |
| 9+ | 5.1 (4.9-5.3) | 5.3 (4.3-6.4) | 21.3 (18.0-25.2) |
| Extrapolated direct medicine costs (/$ million) |  |  |  |
| Patients | $13.8 | $0.7 | $0.5 |
| Government | $12.2 | $2.1 | $5.3 |
| Total | $26.0 | $2.8 | $5.8 |

^†^ Primary indicators only, ^‡^ Calculated by multiple logistic regression, ^||^ age group 1 = 65-74, 2 = 75-84, 3 = 85+ for benzodiazepines in elderly and antipsychotics in dementia and 1 = 18-49, 2=50-64, 3 =65+ for antipsychotic polypharmacy

**Table S3:**,Crude odds ratios of associated patient characteristics in 2016 for each low-value psychotropic prescribing practice

| **Patient characteristics** | **Low-value prescribing practice**^†^ | | |
| --- | --- | --- | --- |
| **Crude** odds ratios  (95% CIs) ^‡^ | Benzodiazepines in elderly | Antipsychotics in dementia | Antipsychotic polypharmacy |
| Sex |  |  |  |
| Male | ref | ref | ref |
| Female | 1.4 (1.3-1.4) | 1.2 (1.1-1.4) | 0.6 (0.6-0.6) |
| Age group |  |  |  |
| 1 | ref | ref | ref |
| 2 | 1.4 (1.4-1.4) | 5.6 (4.9-6.4) | 1 (0.9-1) |
| 3 | 1.8 (1.8-1.9) | 10.1 (8.8-11.7) | 0.5 (0.5-0.6) |
| Comorbidity (RxRisk) Score |  |  |  |
| 0-2 | ref | ref | ref |
| 3-5 | 1.9 (1.8-1.9) | 3.4 (2.9-4) | 3.2 (2.9-3.4) |
| 6-8 | 3.2 (3.1-3.3) | 6 (5.1-7.1) | 4.7 (4.2-5.2) |
| 9+ | 5.4 (5.2-5.6) | 8.6 (7-10.5) | 6.9 (5.9-8.1) |

^†^ Primary indicators only, ^‡^ Calculated by unilogistic regression, ^||^ age group 1 = 65-74, 2 = 75-84, 3 = 85+ for benzodiazepines in elderly and antipsychotics in dementia and 1=18-49, 2=50-64, 3=65+ for antipsychotic polypharmacy

**Table S4:** Prevalence of each low-value practice in 2016 based on primary indicators by sex and age group

| Prevalence (%) | Benzodiazepines in elderly | Antipsychotics in dementia | Antipsychotic polypharmacy |
| --- | --- | --- | --- |
| Sex |  |  |  |
| M | 5.3 | 0.4 | 0.2 |
| F | 7.3 | 0.5 | 0.1 |
| Age group ^†^ |  |  |  |
| 1 | 12.6 | 0.1 | 0.2 |
| 2 | 17.7 | 0.8 | 0.2 |
| 3 | 21.5 | 1.4 | 0.1 |

^†^ Age group 1=65-74, 2=75-84, 3=85+ for benzodiazepines in elderly and antipsychotics in dementia and 1=18-49, 2=50-64, 3=65+ for antipsychotic polypharmacy

a)
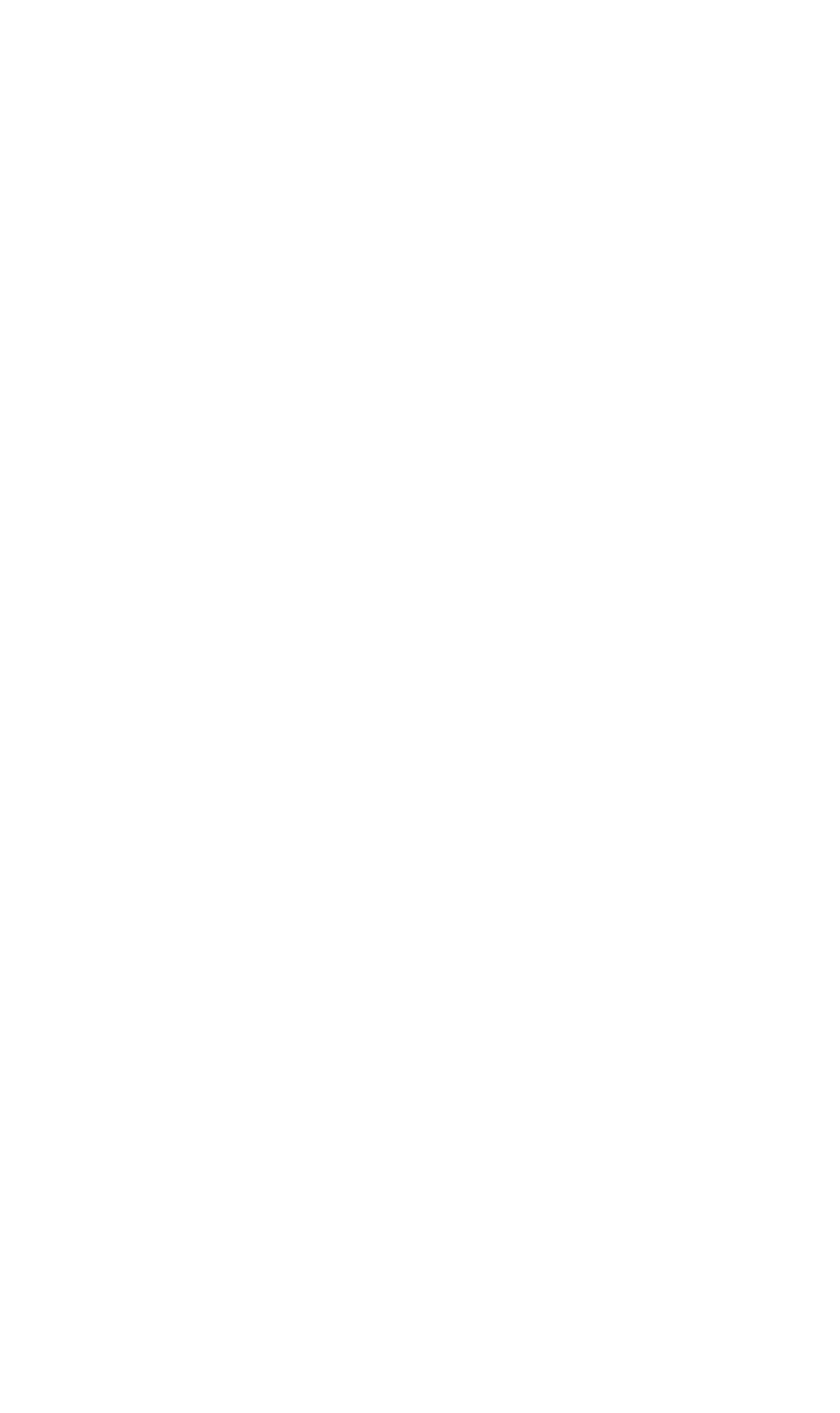


b)
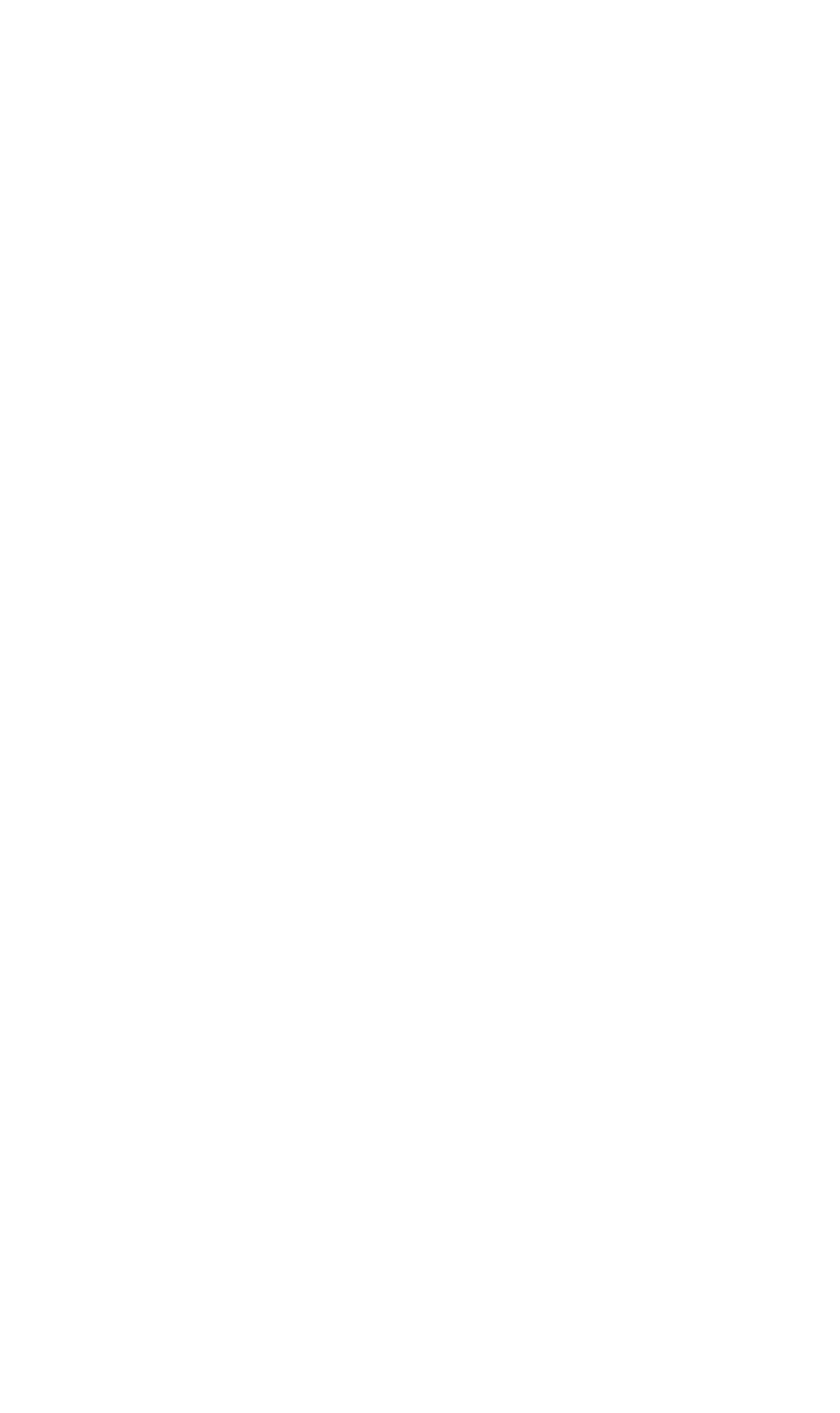


**Figure S1:** Annual rate of low-value prescribing practice indicators; a) Antipsychotic use in dementia (defined by past anti-dementia medicine) b) Antipsychotic polypharmacy (all antipsychotics included)
